# Leptin Modulates the Metastasis of Canine Inflammatory Mammary Adenocarcinoma Cells Through Downregulation of Lysosomal Protective Protein Cathepsin A (*CTSA*)

**DOI:** 10.3390/ijms21238963

**Published:** 2020-11-25

**Authors:** Jin-Wook Kim, Feriel Yasmine Mahiddine, Geon A Kim

**Affiliations:** 1 Department of Theriogenology and Biotechnology, College of Veterinary Medicine, Seoul National University, Gwanak-ro 1, Gwanak-gu, Seoul 08826, Korea; vet_chris@snu.ac.kr (J.-W.K.); yasmini19@snu.ac.kr (F.Y.M.); 2 Department of Theriogenology, Veterinary Medicine Teaching Hospital, College of Veterinary Medicine, Seoul National University, Gwanak-ro 1, Gwanak-gu, Seoul 08826, Korea; 3 Department of Biomedical Laboratory Science, School of Medicine, Eulji University, Daejeon 34824, Korea

**Keywords:** leptin, canine adenocarcinoma, *CTSA*, metastasis, obesity

## Abstract

Canine malignant mammary gland tumors present with a poor prognosis due to metastasis to other organs, such as lung and lymph node metastases. Unlike in human studies where obesity has been shown to increase the risk of breast cancer, this has not been well studied in veterinary science. In our preliminary study, we discovered that leptin downregulated cathepsin A, which is responsible for lysosomal-associated membrane protein 2a (*LAMP2a*) degradation. *LAMP2a* is a rate-limiting factor in chaperone-mediated autophagy and is highly active in malignant cancers. Therefore, in this study, alterations in metastatic capacity through cathepsin A by leptin, which are secreted at high levels in the blood of obese patients, were investigated. We used a canine inflammatory mammary gland adenocarcinoma (CHMp) cell line cultured with RPMI-1640 and 10% fetal bovine serum. The samples were then subjected to real-time polymerase chain reaction, Western blot, immunocytochemistry, and lysosome isolation to investigate and visualize the metastasis and chaperone-mediated autophagy-related proteins. Results showed that leptin downregulated cathepsin A expression at both transcript and protein levels, whereas *LAMP2a*, the rate-limiting factor of chaperone-mediated autophagy, was upregulated by inhibition of *LAMP2a* degradation. Furthermore, leptin promoted *LAMP2a* multimerization through the lysosomal *mTORC2* (mTOR complex 2)/*PH* domain and leucine rich repeat protein phosphatase 1 (*PHLPP1*)/*AKT1* (Serine/threonine-protein kinase 1) pathway. These findings suggest that targeting leptin receptors can alleviate mammary gland cancer cell metastasis in dogs.

## 1. Introduction

Leptin is a peptide hormone produced and secreted mainly by adipose cells, playing a crucial role in reproduction [1], appetite [2], and cancer environment [3]. Previous studies described leptin as a tumor metastasis-promoting factor that induces inflammation [4] and epithelial–mesenchymal transition (EMT), mostly in malignant breast [5,6] and lung cancers [7,8]. Thus, many studies have been conducted to find novel antagonists against the leptin receptor (OBR) [9]. Of these, Allo-aca, a leptin antagonist binding selectively to the C-terminus of OBR without any agonistic effects, has been shown to be effective against triple-negative breast cancer (TNBC) by increasing survival rates [10]. As the blood leptin concentration increases proportionally to the degree of obesity and aging in both humans and dogs [11,12,13,14,15], the regulation of leptin activity is crucial for managing and inhibiting cancer. Given the potential similarity between them, canine mammary gland tumors (CMTs) are considered as a model for investigating human breast cancer [16]. Similar to women, mammary gland tumors are the most common tumor in domestic intact bitches, and approximately half of them are diagnosed as malignant [11]. Canine inflammatory mammary carcinoma (cIMC) is a fast-growing, malignant form that shows poor prognosis (mean survival time of 25 days) because of its high incidence of metastasis to regional lymph nodes [17], which has risk factors that are similar to those of human breast cancers (e.g., age, progesterone, obesity) [18,19].

It has been suggested that leptin upregulates autophagy and promotes lysosomal degradation of long-lived proteins in adipose cells [20] and MCF-7 cells [4]. Lysosomal activity is closely related to autophagy, especially chaperone-mediated autophagy (CMA), a selective form of autophagy targeting substrate proteins bearing KFERQ-motifs (e.g., *GAPDH*) [21]. It is involved in cellular homeostasis and is highly expressed in cancer, promoting proliferation, anti-apoptosis, and metastasis [22,23]. The multimerization of lysosomal-associated membrane protein 2a (*LAMP2a*) on the lysosomal membrane is crucial for binding with the chaperone protein *HSP70 (Heat shock protein 70)* through the lysosomal *mTORC2* (mTOR complex 2)/PH domain and leucine rich repeat protein phosphatase 1 (*PHLPP1*)/*AKT1* (Serine/threonine-protein kinase 1) pathway [24]. Cuervo and her colleagues demonstrated that the lysosomal enzyme cathepsin A (*CTSA*) regulates the half-life of *LAMP2a*, the rate-limiting factor for CMA, through its degradation [25]. Mutations in the *CTSA* gene cause the lysosomal storage disorder galactosialidosis both in humans and dogs [26,27]. However, the association between leptin and CMTs has not yet been described. Moreover, although previous studies have described the relationship between cathepsins and leptin [28,29], *CTSA* has not been extensively studied in cancer metastasis. Therefore, in this study, we mainly focused on the alteration of metastasis of canine inflammatory mammary adenocarcinoma (CHMp) cells with leptin (with or without Allo-aca) and *CTSA* and further evaluated the alteration of CMA activity.

## 2. Results

### 2.1. Leptin Downregulated CTSA and Upregulated LAMP2a in CHMp Cells

**Figure 1:**
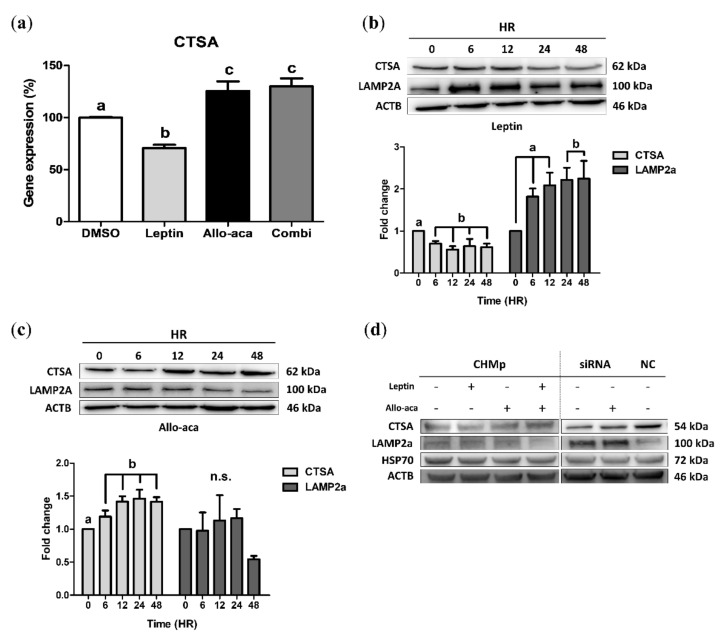
Leptin downregulated the cathepsin A (*CTSA*) gene in canine inflammatory mammary adenocarcinoma (CHMp) cells. (**a**) *CTSA* gene expression in CHMp cells treated with leptin and Allo-aca was evaluated using real-time PCR. (**b**,**c**) Western blot analysis of time-course *CTSA*/lysosomal-associated membrane protein 2a (*LAMP2a*) alteration induced by leptin (**b**) and Allo-aca (**c**) treatment. (**d**) Western blot analysis of chaperone-mediated autophagy (CMA)-related genes. Cells were incubated with leptin and/or Allo-aca for 24 h, and *CTSA* small interfering ribonucleic acid (siRNA) was transfected 24 h before chemical treatment. All graphs are visualized as mean ± standard error of the mean (SEM) with at least three replicates. The column bars with different alphabetical letters indicate significant difference among groups (*p* < 0.05). Combi: combination of leptin and Allo-aca; NC: negative control siRNA.

To determine the optimal concentration of leptin and Allo-aca, we introduced leptin into CHMp cells. The 3-(4,5-dimethylthiazol-2-yl)-2,5-diphenyltetrazolium bromide (MTT) assay showed that 6 nM leptin had a cell proliferation effect, while Allo-aca did not show this effect, even at 100 nM (the highest concentration) (Figure S1a,b). Interestingly, *CTSA* mRNA was significantly decreased with 12 nM leptin treatment and increased at the lowest concentration (1 nM) with Allo-aca (Figure S1c,d). Thus, we treated cells with 12 nM leptin and/or 100 nM Allo-aca to maximize its antagonistic effect. Real-time PCR analysis showed that gene expression was upregulated following treatment with Allo-aca, disregarding the presence of leptin (Figure 1a). Subsequently, the time-course immunoblot of *CTSA* and *LAMP2a* showed that leptin gradually downregulated *CTSA* from 6 h while significantly upregulating *LAMP2a* after 24 h (Figure 1b). Conversely, Allo-aca upregulated *CTSA* after 6 h of treatment, but no significance was observed in *LAMP2a* (Figure 1c).

As shown in Figure 1d, Allo-aca antagonized the effect of leptin in CHMp cells, resulting in decreased *LAMP2a* expression. To elucidate the role of leptin in CMA, we transfected CHMp cells with *CTSA* small interfering ribonucleic acid (siRNA) (Figure S2). Immunoblot analysis showed that Allo-aca upregulated *CTSA* in the transfected cells, but the expression of *LAMP2a* was not significantly altered.

### 2.2. Leptin Promoted Cell Proliferation in CHMp Cells

**Figure 2:**
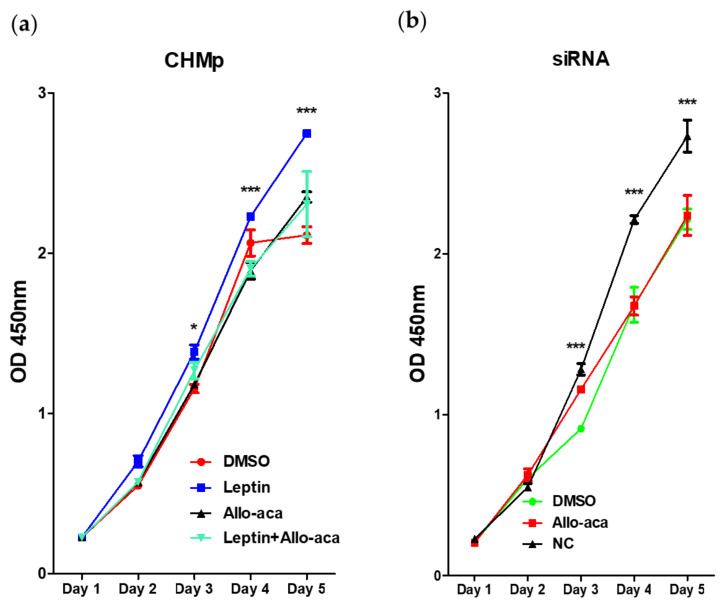
Cell proliferation assay. Quantitative measurement was determined by 3-(4,5-dimethylthiazol-2-yl)-2,5-diphenyltetrazolium bromide (MTT) assay. Five thousand cells per well were seeded and cultured for 5 days in a 37 °C, 5% CO_2_ incubator. (**a**) Leptin significantly promoted cancer cell proliferation from day 3, whereas Allo-aca inhibited its effect in CHMp cells. (**b**) Allo-aca treatment on *CTSA* siRNA-transfected cells did not affect cell proliferation. However, it seemed that the knockdown of *CTSA* decreased cell proliferation compared to the negative control group. The results were expressed as mean ± SEM. * *p* < 0.05, *** *p* < 0.001.

Cell proliferation assay showed that the proliferation capacity of CHMp cells was significantly increased with 12 nM leptin treatment, and Allo-aca antagonized its effect (Figure 2a), resulting in a significantly lower proliferation index. Moreover, Allo-aca alone did not show a negative effect on cell proliferation, as no significant difference was observed between the control and Allo-aca groups. In agreement with the previous results, we also found that the knockdown of *CTSA* using siRNA inhibited cell proliferation, and that Allo-aca did not affect cell proliferation in transfected cells (Figure 2b).

### 2.3. Leptin Stimulated EMT in CHMp Cells

**Figure 3:**
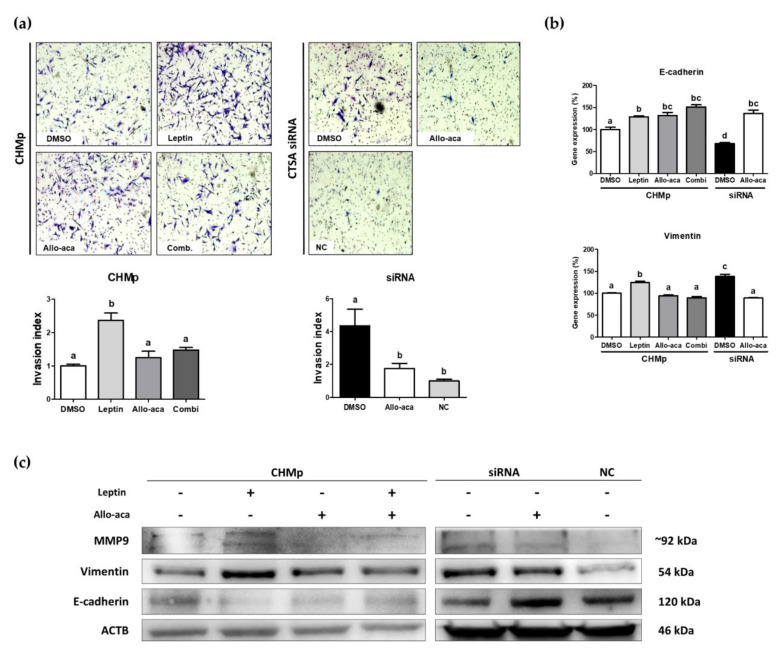
Leptin stimulated epithelial–mesenchymal transition (EMT) in CHMp cells. (**a**) Matrigel invasion assay and calculated invasion index of CHMp and siRNA-transfected cells. Invaded cells were counted under light microscopy (40×) using ImageJ software. (**b**) Comparison of EMT-related gene (*E-cadherin* and *vimentin*) expression using real-time PCR analysis among experimental groups. (**c**) Western blot analysis of tumor invasion-related genes. All graphs are visualized as mean ± SEM with at least three replicates. The column bars with different alphabetical letters indicate significant difference among groups (*p* < 0.05). Combi: combination of leptin and Allo-aca; NC: negative control siRNA.

To investigate the role of leptin in canine mammary adenocarcinoma progression, we introduced leptin and Allo-aca into CHMp cells. Matrigel invasion assay (Figure 3a) showed that the invasion capacities of CHMp cells were significantly increased by leptin treatment, while those of Allo-aca-treated cells (including the combination group) remained the same as the control group. In addition, siRNA-transfected cells showed significantly increased invasion capacities, but these were significantly reduced by treatment with Allo-aca.

Real-time PCR of EMT-related genes showed that both *E-cadherin* and *vimentin* were significantly altered by leptin treatment. Allo-aca significantly upregulated *E-cadherin*, a cell adhesion factor, in both CHMp cells and transfected cells. However, leptin and siRNA significantly upregulated *vimentin*, whereas its effect was inhibited by Allo-aca (Figure 3b).

To validate whether leptin alters EMT genes at the translational level, we also performed Western blot analysis (Figure 3c). The overall tendencies were matched with mRNA expression, but leptin and knockdown of *CTSA* significantly upregulated *MMP9* (matrix metalloproteinase 9), a biomarker of tumor invasion and metastasis [30]. In addition, Allo-aca downregulated *vimentin* and *MMP9* and upregulated *E-cadherin* in both cell types. Taken together, these results suggest that leptin and subsequent *CTSA* downregulation stimulate tumor invasion and metastasis via activation of the EMT process.

### 2.4. Leptin Delayed the Degradation of LAMP2a Through Downregulation of CTSA

**Figure 4:**
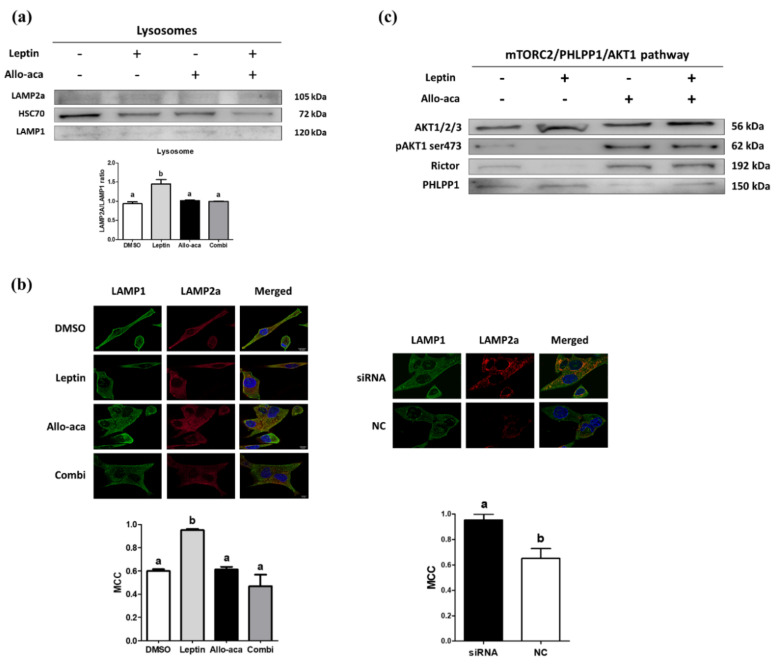
Leptin induced a higher *LAMP2a*/*LAMP1* ratio in lysosomal membranes. (**a**) Western blot analysis of lysosomes isolated from CHMp cells targeting CMA-associated lysosomal proteins. The results showed that the *LAMP2a*/*LAMP1* ratio was increased with leptin treatment and Allo-aca acted as a leptin receptor (OBR) antagonist. Then, the samples were subjected to SDS-PAGE. (**b**) Leptin and Allo-aca-treated CHMp cells and *CTSA* siRNA were visualized with confocal microscopy with immunofluorescence technique. The colocalization ratio was measured by Fiji software developed by Schindelin and colleagues [31] and analyzed on the basis of Mander’s colocalization coefficient (MCC). The results were in concordance with the lysosome immunoblot experiments. (**c**) Western blot analysis of the lysosomal *mTORC2*/PH domain and leucine rich repeat protein phosphatase 1 (*PHLPP1*)/*AKT1* pathway in lysosomes isolated from CHMp cells. *pAKT1(ser473)* and rictor were inhibited with leptin treatment, but *PHLPP1* was stimulated. All graphs are visualized as mean ± SEM with at least three replicates. The column bars with different alphabetical letters indicate significant difference among groups (*p* < 0.05). Combi: combination of leptin and Allo-aca; NC: negative control siRNA.

To specifically investigate the alteration of lysosomal proteins, we isolated lysosome fractions from CHMp cells. We found that the *LAMP2a*/*LAMP1* ratio increased with leptin treatment, and no significant differences were observed among the DMSO (dimethyl sulfoxide), Allo-aca, and combination groups (Figure 4a). In addition, HSC70 (Heat shock cognate 71kDa protein), *LAMP2a*, and *LAMP1* colocalization tests analyzed with Mander’s colocalization coefficient (MCC) demonstrated that leptin and knockdown of *CTSA* increased the ratio, while Allo-aca showed no agonistic effect and an antagonistic effect on leptin (Figure 4b). On the basis of these results, we can assume that leptin and subsequent downregulation of *CTSA* are possibly correlated with higher CMA activity.

### 2.5. Leptin May Promote Lysosomal LAMP2a Multimerization Through the mTORC2/PHLPP1/AKT1 Pathway

**Figure 5:**
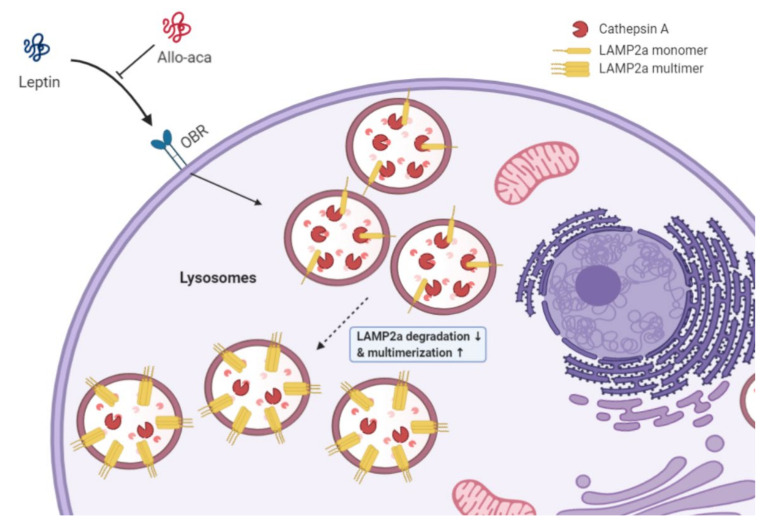
Schematic images describing the summarized results in this study. Leptin downregulated *CTSA* and, following delayed *LAMP2a* degradation, resulted in CMA activation and stimulation of *LAMP2a* multimerization through the *mTORC2*/*PHLPP1*/*AKT*1 pathway.

To further investigate the role of leptin in the regulation of CMA, we analyzed the lysosomal kinase/phosphatase complex. We found that leptin downregulates rictor, a subunit of the *mTORC2* complex, and upregulates *PHLPP1*, a serine/threonine phosphatase, inhibiting the action of *AKT*1 via phosphorylation of *pAKT1(ser473)*. Conversely, similar to the previous results, Allo-aca antagonized these effects regardless of the presence of leptin (Figure 4c). Thus, we can suggest that leptin promotes the multimerization of *LAMP2a* in lysosomal membranes through the activation of the *mTORC2*/*PHLPP1*/*AKT*1 pathway and that Allo-aca can prevent the formation of *LAMP2a* multimers (Figure 5).

## 3. Discussion

In this study, we investigated the concurrent effects of leptin on the lysosomal enzyme *CTSA* and the following effects on tumor cell proliferation and invasion capacity of CHMp, a canine mammary adenocarcinoma cell line. In our preliminary study, we found that *CTSA* gene expression was significantly downregulated and enhanced cell viability with 12 nM leptin treatment, which was reversed with the OBR antagonist Allo-aca. Interestingly, Allo-aca itself could also inhibit EMT and metastasis in both cell types. Moreover, the increased expression of *MMP9* indicates a higher chance of metastasis to other organs [32]. To determine the effect of leptin on *CTSA*, we applied *CTSA* siRNA to knock down its expression in CHMp cells. In addition, we also found that leptin promotes tumor cell growth, which is significant in other control and experimental groups in the time-course cell proliferation assay. Interestingly, the knockdown of *CTSA* did not promote tumor cell proliferation but rather inhibited it. As patients with galactosialidosis typically show lower body weight, we suspect that higher *LAMP2a* activity due to delayed degradation is responsible for a slower proliferation rate [25]. Moreover, Allo-aca inhibited leptin-induced cell proliferation in the co-treatment group but did not inhibit the growth itself, and no alteration was observed in transfected cells. Therefore, we focused on utilizing leptin and Allo-aca to investigate the role of *CTSA* in CHMp cells.

We subsequently investigated leptin as a therapeutic target for preventing mammary cancer metastasis in dogs and identified it as an endogenous *LAMP2a* activator by modulating its stability and promoting multimerization. An in vitro invasion assay showed that leptin and knockdown of *CTSA* promoted CHMp cell invasion by upregulating *vimentin* and metallo-proteinase 9 (*MMP9*), and downregulating *E-cadherin*. In addition, supplementation of Allo-aca antagonized the effect of leptin on EMT even without leptin co-treatment. *E-cadherin*, a transmembrane epithelial cell marker, is considered a prognostic factor in both human and dog mammary gland cancer [33,34]. Its decreased expression represents upregulated cancer invasion ability and poor prognosis, causing EMT progression together with increased expression of *vimentin*, a mesenchymal cell marker. The radical knockdown of *CTSA* also induced EMT, similar to leptin treatment. On the basis of the ability to induce EMT, we found leptin and its ability to downregulate *CTSA* to promote the migration and invasiveness of CHMp cells and upregulate metastasis-related genes, which shows poor prognosis for mammary gland tumor patients. In addition, we also found that Allo-aca antagonized leptin in CHMp cells but also reversed the consequences of the knockdown of *CTSA* and further showed anti-EMT effects. Therefore, these results indicate that leptin may also induce metastasis in canine mammary adenocarcinoma cells and that Allo-aca seems to have anti-metastatic properties retrieving *CTSA* expression, which prolongs the half-life of *LAMP2a*.

It has been suggested that the knockdown of *LAMP2a*, which induces lower CMA, reduces the metastatic capacity of lung cancer cells [22]. In other words, the level of CMA in cancer cells is associated with cancer metastasis. In agreement with the previous study, the upregulation of *LAMP2a* through knockdown or leptin treatment in CHMp cells resulted in enhanced invasion and metastasis. These alterations were prevented with Allo-aca treatment and reduced *LAMP2a* ratio normalized by *LAMP1* (*LAMP2a*/*LAMP1* ratio) significantly. That is, the prolonged *LAMP2a* half-life results in a higher level of *LAMP2a* on the lysosomal membrane. Moreover, leptin stimulated *PHLPP1*, thus showing inhibitory effects on *AKT*. *PHLPP1* is known to inhibit CMA activity through *AKT* dephosphorylation. In addition, phosphorylated *AKT*1 ser473 (p*AKT*1(ser473)) is lower in CMA-active lysosomes in starved animals [24]. Together with these findings, we conclude that leptin also promotes CMA activity through *LAMP2a* multimerization (Figure 5). Moreover, Allo-aca can offset its effects in CHMp cells by blocking OBR.

Higher levels of *LAMP2a* in cancers play important roles in cell survival and their microenvironment. Therefore, previous studies have focused on *LAMP2a* as a therapeutic target. However, specific manipulation of one specific gene or protein using RNAi (RNA interference) can result in unpredictable side effects such as RNAi off-target effects and carrier-mediated toxicity [35,36], which can be rather fatal for veterinary patients. Therefore, through Allo-aca treatment, which can offset the CMA-stimulating effect of leptin demonstrated in this study, we suggest the possibility of inhibiting metastasis with relatively few side effects in the treatment of cIMCs. However, the fact that Allo-aca itself can enter the blood–brain barrier must be improved because it can stimulate appetite and weight gain.

Although canine inflammatory mammary adenocarninoma has not been studied much, in terms of the subject, CHMp is a relatively well-phenotyped cell line and thus can represent the alterations of cIMCs shown in the study with leptin and Allo-aca. However, the single use of CHMp cell line cannot represent the definitive physiology of all types (epithelial, myoepithelal, mesenchymal) of CMTs because the cell line is of an epithelial origin. Moreover, the lack of direct confirmation of CMA activity as a substrate and the inability to quantitatively analyze *LAMP2a* multimerization using blue native PAGE due to the absence of suitable antibodies are limitations in this study. Further studies on samples from mammary adenocarcinoma patients and their retrospective studies are needed to assess clinical validity of Allo-aca as an anti-metastases agent. 

## 4. Materials and Methods

### 4.1. Cell Lines and Cell Culture Methods

The CHMp cell line was generously donated by Professor So Young Lee from the Department of Veterinary Pharmacology, College of Veterinary Medicine, Seoul National University, Seoul, Korea. The cells were originally isolated from the primary lesions of 12-year-old mixed-breed female inflammatory mammary adenocarcinoma and established as a cell line by Professor Nobuo Sasaki from the University of Tokyo [37]. The cells were cultured in RPMI-1640 supplemented with 10% charcoal-dextran treated fetal bovine serum (both from Gibco, Waltham, MA, USA), and 0.5% gentamicin (Merck), in a 5% carbon dioxide 37 °C incubator.

### 4.2. Chemicals and Antibodies

Canine leptin was purchased from Peprotech (Rocky Hill, NJ, USA), and Allo-aca (H-alloThr-Glu-Nva-Val-Ala-Leu-Ser-Arg-Aca-NH_2_), developed by Otvos et al. [38], was synthesized by Koma Biotechnology (Seoul, Korea). DMSO was used as their solvent and the control group. Co-treatment of leptin and Allo-aca is described as Combi in this study. All other chemicals were purchased from Sigma-Aldrich (St. Louis, MO, USA), unless otherwise stated. The antibodies used in this study are summarized in Table 1.

### 4.3. Reverse siRNA Transfection

Cathepsin A siRNA (*CTSA* siRNA) was transfected using Lipofectamine RNAiMAX (Invitrogen, Waltham, MA, USA). Before seeding the cells in 6-well dishes, we diluted 50 pmol of *CTSA* siRNA in 500 µL Opti-MEM (Gibco, Waltham, MA, USA) with the addition of 7 µL of Lipofectamine RNAiMAX (Invitrogen, Waltham, MA, USA). After 20 min of incubation at room temperature, 300,000 CHMp cells were seeded and incubated for 24 h and then used for further experiments. The siRNA sequences are listed in Table S1.

### 4.4. Cell Proliferation Assay

Five thousand cells per well were seeded in a 96-well dish with phenol red-free RPMI-1640 and 10% FBS (Fetal bovine serum) and treated with 12 nM leptin and 100 nM Allo-aca for 5 days to exclude the interference of phenol red. Each day, the whole medium was discarded and washed with phosphate-buffered saline (PBS). Then, 10 µL of 12 mM MTT (3-(4,5-dimethylthiazol-2-yl)-2,5-diphenyltetrazolium bromide; Invitrogen, Waltham, MA) in 100 µL phenol red-free RPMI-1640 (Gibco, Waltham, MA, USA) were added and incubated for 4 h at 37 °C to form the formazan crystals. After incubation, 85 µL of MTT-containing media was discarded, and 80 µL of DMSO was added as a solvent to dissolve the formazan for 10 min at 37 °C following horizontal shaking for 30 min in the dark. The technical replicates of each variable were repeated 4 times. The samples were analyzed at 450 nm using a Tecan Sunrise Microplate Reader (Tecan, Männedorf, Switzerland).

### 4.5. Cell Invasion Assay

The cultured cells were treated with non-enzymatic cell dissociation solution (Sigma-Aldrich, St. Louis, MO, USA) for 5 min to detach cells without damaging cell surface receptors. Suspended cells were subjected to a cell invasion assay using 8 µm BioCoat Matrigel invasion chambers (Corning, Bedford, MA, USA). Cells (2 × 10^5^) were seeded in each well with RPMI-1640 + 10% FBS and incubated for 22 h. After incubation, the upper membrane was swabbed with sterile cotton swabs to remove non-invading cells and stained with Diff-Quick solution (Sysmax, Tokyo, Japan). The invaded cells were counted under a 40 × bright microscope and counted with ImageJ software (National Institute of Health, Bethesda, MD, USA).

### 4.6. RNA Extraction and Real-time Quantitative PCR Analysis (RT-qPCR)

After 24 h of incubation with the addition of leptin and/or Allo-aca, the cells were collected for RNA extraction. We used an easy-spin Total RNA Extraction Kit, and complementary DNA (cDNA) synthesis was performed using the Maxime RT PreMix Kit (both kits from Intron Biotechnology, Gyeonggi, Korea) according to the manufacturer’s instructions. For RT-qPCR analysis, we used 2× SYBR^®^ Green PCR Master Mix (Applied Biosystems, MA, USA) as a probe, and the primer sequences are listed in Table 2. Amplification was performed using the StepOnePlus Real-Time PCR System (Applied Biosystems, MA, USA) with reactions containing 50 ng cDNA, 10 pmol of forward and reverse primers, 10 μL of SYBR green premix, and nuclease-free water in each well. The thermocycling protocol was 40 cycles of 95 °C for 10 s, 60 °C for 20 s, and 72 °C for 40 s. All reaction cycles were conducted in a MicroAmp Optical 96-Well Reaction Plate (Applied Biosystems, MA, USA). To compare mRNA expression based on leptin and Allo-aca treatment in CHMp cells, we normalized mRNA expression to the housekeeping gene β-actin (ΔCt), and the relative amount of each transcript was quantified in at least 3 replicates using the following equation: relative quantity (R) = 2^−(ΔCt sample−ΔCt control)^.

### 4.7. Immunofluorescence Analysis

The cells were seeded in 8-well SPL Cell Culture (SPL Life Science, Gyeonggi, Korea) at a density of 5000 cells per well. After 6 h of incubation, cells were fixed with 4% paraformaldehyde and washed with ice-cold phosphate-buffered saline (PBS). The cells were then permeabilized with 0.1% Triton X-100 diluted in PBS for 10 min. One percent bovine serum albumin (BSA) in PBS-Tween 20 was used for blocking for 1 h, and primary antibodies against *LAMP1* (diluted 1:200 in PBS-T with 1% BSA) were incubated overnight at 4 °C. The next day, goat anti-rabbit IgG (Immunoglobulin G) H&L Alexa Fluor 488 antibody against *LAMP1* primary antibodies was added for 1 h at room temperature in the dark. For the anti-*LAMP2a* antibody, the antibody was manually conjugated with Alexa Fluor 647 Conjugation Kit-Lightning-Link (Abcam, Cambridge, United Kingdom) to visualize colocation of *LAMP1* and *LAMP2a* despite the same antibody origin, and incubated overnight in the dark. The slides were then washed with ice-cold PBS and then mounted with Vectashield Antifade Mounting Medium (Vector Laboratories, Peterborough, United Kingdom). All images were obtained using a Ti2-E confocal microscopy system (Nikon, Tokyo, Japan). The negative control images are attached in Figure S3.

### 4.8. Lysosome Isolation and Immunoblot

CHMp cells were cultivated in 148.2 mm dishes at a density of 5 × 10^6^ and then treated with 0.01% DMSO (control), leptin (12 nM), Allo-aca (100 nM), or combination of leptin and Allo-aca. After treatment with chemicals, the cells were collected with disposable cell scrapers. Lysosomes were then isolated from cells using the Minute Lysosome Isolation Kit (Invent Biotechnologies, Plymouth, MN, USA) on the basis of the spin-column method. Approximately 1.0 × 10^8^ cells were subjected to lysosome isolation. The isolated lysosomes were resuspended in RIPA (Radioimmuno-precipitation assay) buffer (Thermo Fisher Scientific, Waltham, MA, USA) supplemented with a protein inhibitor cocktail and phosphatase inhibitor to prevent protein degradation, blocking the activity of phosphatases, and were centrifuged at 16,000× *g* for 30 min. The supernatant was transferred to a new 1.5 mL tube and subjected to protein quantification using the Pierce BCA Protein Assay Kit (Thermo Fisher Scientific, Waltham, MA, USA). Lysosome lysates (10 μg) were loaded onto 12% Mini-PROTEAN TGX Precast gels. After transfer and blocking, the membranes were incubated with primary overnight and developed with HRP (Horseradish peroxidase)-conjugated secondary antibodies.

### 4.9. Western Blot Analysis

For time-course investigation of *CTSA* and *LAMP2a*, we collected the samples at 0, 6, 12, 24, and 48, h of incubation with leptin and Allo-aca to assess the alteration of CMA-related factors. For EMT pathway investigation, the samples were collected at 24 h of incubation. The samples were lysed with PRO-PREP Protein Extraction Solution (Intron Biotechnology, Gyeonggi, Korea), and their protein concentration was measured by Bradford assay. For SDS-PAGE, 5 µg of each sample was loaded onto each well of 12% Mini-PROTEAN TGX Precast Gel (Bio-Rad, Hercules, CA, USA) and paged at 100 V for 1 h 30 min. The samples were then transferred to 0.45 µm methanol-activated polyvinylidene fluoride (PVDF) membranes (Millipore, Burlington, MA, USA) at 350 mA for 1 h 10 min. The membranes were blocked with 5% skim milk solution and washed 3 times each with TBS-T for 10 min, followed by primary antibody incubation overnight. The next day, the membranes were incubated with HRP-conjugated secondary antibody for 1 h. For primary antibodies, we used rabbit anti-*LAMP1*, *LAMP2a*, cathepsin A (PPCA), mouse anti-*HSP70*, β-actin, *PHLPP1*, *AKT*1, p*AKT*1(ser473), and rat anti-*E-cadherin* (*CDH1*).

For time-course investigation of *CTSA* and *LAMP2a*, we collected the samples at 0, 6, 12, 24, and 48, h of incubation with leptin and Allo-aca to assess the alteration of CMA-related factors. For EMT pathway investigation, the samples were collected at 24 h of incubation. The samples were lysed with PRO-PREP Protein Extraction Solution (Intron Biotechnology, Gyeonggi, Korea), and their protein concentration was measured by Bradford assay. For SDS-PAGE, 5 µg of each sample was loaded onto each well of 12% Mini-PROTEAN TGX Precast Gel (Bio-Rad, Hercules, CA, USA) and paged at 100 V for 1 h 30 min. The samples were then transferred to 0.45 µm methanol-activated polyvinylidene fluoride (PVDF) membranes (Millipore, Burlington, MA, USA) at 350 mA for 1 h 10 min. The membranes were blocked with 5% skim milk solution and washed 3 times each with TBS-T for 10 min, followed by primary antibody incubation overnight. The next day, the membranes were incubated with HRP-conjugated secondary antibody for 1 h. For primary antibodies, we used rabbit anti-*LAMP1*, *LAMP2a*, cathepsin A (PPCA), mouse anti-*HSP70*, β-actin, *PHLPP1*, *AKT*1, p*AKT*1(ser473), and rat anti-*E-cadherin* (*CDH1*).

### 4.10. Statistical Analysis

The raw data were analyzed with GraphPad Prism 8.0 (San Diego, CA, USA) with one-way ANOVA followed by Bonferroni’s test for further analysis. The Western blot and immunocytochemistry images were analyzed with ImageJ software (National Institute of Health (NIH), Bethesda, MD, USA) according to NIH guidelines.

## 5. Conclusions

From our findings, we suggest that leptin, which increases with age and obesity in dogs, can stimulate mammary gland cancer metastasis by elongating the half-life and multimerization of *LAMP2a*. We also demonstrated that Allo-aca can reverse the promotion of EMT and invasiveness of CHMp cells treated with leptin through selective pairing to OBR without any agonistic effects. These findings provide evidence that leptin receptor antagonists are a therapeutic and prophylactic option for cIMCs without unwanted side effects.

## Figures and Tables

**Table 1:** Antibodies used in this study.

Target	Catalog Number	Host	Clone	Dilution	Manufacturer
*LAMP2a*	ab18528	Rabbit	Polyclonal	1:1000	Abcam
*LAMP1*	ab24170	Rabbit	Polyclonal	1:1000	Abcam
*HSP70*	ab53496	Mouse	Monoclonal	1:1000	Abcam
*VIM*	ab1316	Mouse	Monoclonal	1:400	Abcam
*MMP9*	ab38898	Rabbit	Polyclonal	1:1000	Abcam
*pAKT1(ser473)*	ab81283	Rabbit	Monoclonal	1:5000	Abcam
*ACTB*	ab8227	Mouse	Monoclonal	1:5000	Abcam
*CDH1*	14-3249-82	Rat	Monoclonal	1:250	Invitrogen
*AKT*1	sc-135829	Mouse	Monoclonal	1:1000	SCBT
*PHLPP1*	sc-390129	Mouse	Monoclonal	1:500	SCBT
mTOR	sc-517464	Mouse	Monoclonal	1:200	SCBT
***CTSA***	139645	Rabbit	Polyclonal	1:1000	US Biological

**Table 2:** Primers and their sequences used in this study. ^1^ *CDH1* is also known as *E-cadherin*.

Gene	GenBank Accession No.	Primer Sequences	Product Size (bp)
*CTSA*	NM_001109915.1	F: 5′-CAG ACC CAC TGC TGT TCT CA-3′	56
		R: 5′-CTG CAG ATT TGT CAC GCA TT-3′	
		R: 5′-GAG TAC TTC AGG GCC GTC AG-3′	
*VIM*	NM_001287023.1	F: 5′-CCG ACA GGA TGT TGA CAA TG-3′	116
		R: 5′-GCT CCT GGA TTT CCT CAT CA-3′	
*CDH1* ^1^	NM_001287125.1	F: 5′-AAT GAC CCA GCT CGT GAA TC-3′	108
		R: 5′-CAC CTG GTC CTT GTT CTG GT-3′	
*ACTB*	NM_001195845.2	F: 5′-GCG CAA GTA CTC TGT GTG GA-3′	65
		R: 5′-ACA TTT GCT GGA AGG TGG AC-3′	

F: Forward; R: Reverse

## Supplementary Materials

Supplementary Materials can be found at https://www.mdpi.com/1422-0067/21/23/8963/s1.

## Abbreviations

CMT	Canine mammary gland tumor
cIMC	Canine inflammatory mammary carcinoma
*CTSA*	Lysosomal protective protein cathepsin A (PPCA)
*LAMP1*	Lysosomal-associated membrane protein 1
*LAMP2a*	Lysosomal-associated membrane protein 2a
CMA	Chaperone-mediated autophagy
OBR	Leptin receptor
*PHLPP1*	PH domain and leucine rich repeat protein phosphatase 1
*ACTB*	Beta-actin
*VIM*	*Vimentin*
*CDH1*	*E-cadherin*
siRNA	Small interfering ribonucleic acid
cDNA	Complementary deoxyribonucleic acid
mRNA	Messenger ribonucleic acid
RNAi	RNA interference
DMSO	Dimethyl sulfoxide
