# Supplementary material for: Leptin Modulates the Metastasis of Canine Inflammatory Mammary Adenocarcinoma Cells Through Downregulation of Lysosomal Protective Protein Cathepsin A (CTSA)
Source: Int J Mol Sci. 2020 Nov 25;21(23):8963. doi: 10.3390/ijms21238963 (PMC7728357; doi:10.3390/ijms21238963)
Supplement: Supplementary file 1 [file ijms-21-08963-s001.pdf]

Supplementary materials

Jin-Wook Kim, Feriel Yasmine Mahiddine, Geon A Kim

Table S1. CTSA siRNA sequences used in this study. The selected siRNA was marked as **bold**

siRNA	Accession No.	Sequences
CTSA_siRNA_1	NM_001109915.1	CGUGACAAAUCUGCAGGAA
		UUCCUGCAGAUUUGUCACG
**CTSA_siRNA_2**		CACAGAAAUACCGGAUCUU
		AAGAUCCGGUAUUUCUGUG
CTSA_siRNA_3		UGUUCUCAGAACAAGUGUA
		UACACUUGUUCUGAGAACA

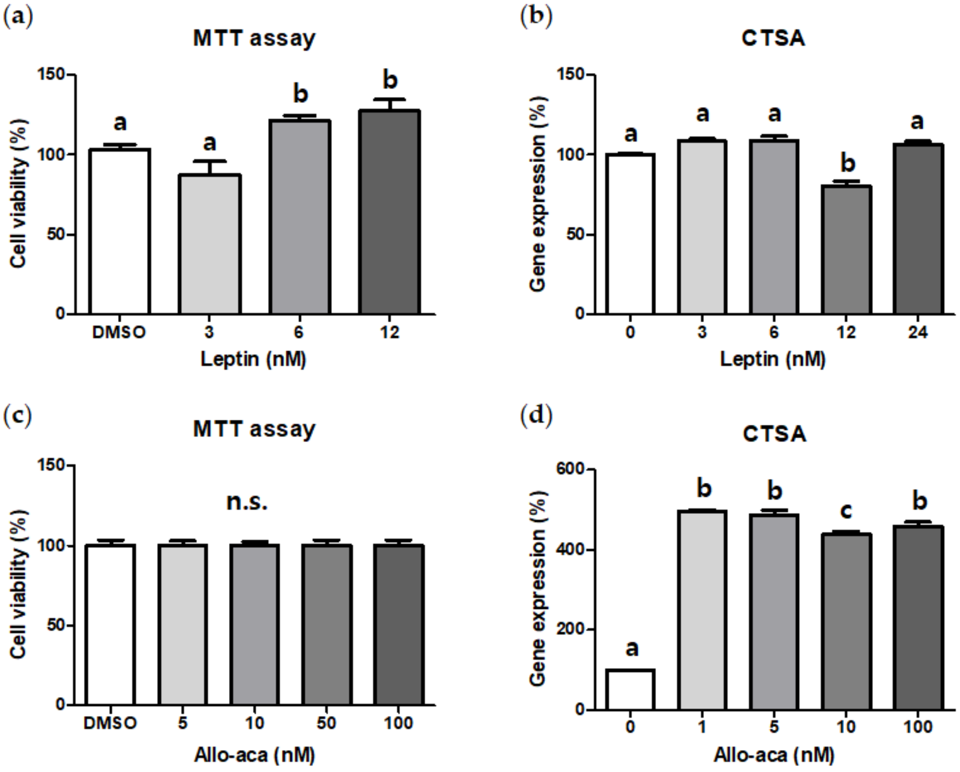

Figure S1. Preliminary studies on determining optimal concentration of leptin and Allo-aca. (**a,b**) MTT assay shows leptin significantly increases cell number from 6 nM and downregulates CTSA gene at 12 nM dose. (**c,d**) Although, Allo-aca did not alter cell number among different concentrations nor show any cytotoxic effects, it significantly upregulates CTSA gene from the lowest dose. * n.s.;not significant.

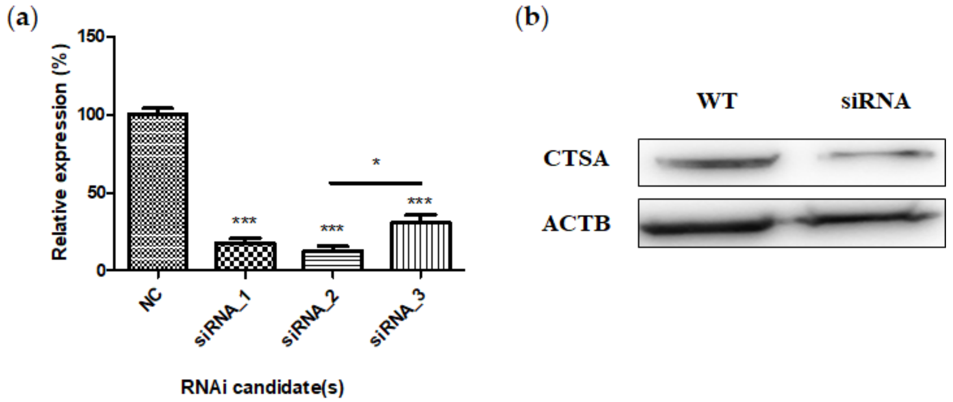

Figure S2. siRNA-induced knockdown of cathepsin A in CHMp cell line. Real-time reverse-transcription quantitative PCR(RT-qPCR) and western blot analysis confirm the knockdown of cathepsin A (CTSA) in CHMp cells. We used Lipofectamine RNAiMAX for transfection and followed reverse transfection protocol provided by the manufacturer. The cells were collected 24 hours after transfection and immediately processed for the analysis. (**a**) siRNA_2 showed the highest efficacy among 3 candidates. (**b**) Western blot analysis confirmed the downregulated expression of CTSA in siRNA_2 treated group. NC;Negative control (* $p < 0.5$, *** $p < 0.001$)

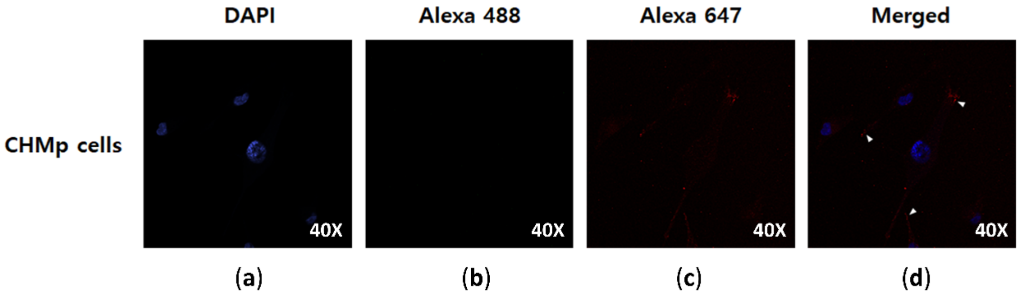

Figure S3. Confocal microscopy images of negative control CHMp cells. (**a**) DAPI stained CHMp cells; (**b**) Negative control image of Alexa 488-conjugated secondary antibody. (**c**,**d**) Negative control image of Alexa 647-conjugated LAMP2a antibodies. The white arrow-heads indicate the location of LAMP2a only. The images are captured under 40X confocal microscope.
